# An “Escape Clock” for Estimating the Turnover of SIV DNA in Resting CD4+ T Cells

**DOI:** 10.1371/journal.ppat.1002615

**Published:** 2012-04-05

**Authors:** Jeanette Reece, Janka Petravic, Mehala Balamurali, Liyen Loh, Shayarana Gooneratne, Rob De Rose, Stephen J. Kent, Miles P. Davenport

**Affiliations:** 1 Department of Microbiology and Immunology, University of Melbourne, Parkville, Victoria, Australia; 2 Centre for Vascular Research, University of New South Wales, Sydney, New South Wales, Australia; Emory University, United States of America

## Abstract

Persistence of HIV DNA presents a major barrier to the complete control of HIV infection under current therapies. Most studies suggest that cells with latently integrated HIV decay very slowly under therapy. However, it is much more difficult to study the turnover and persistence of HIV DNA during active infection. We have developed an “escape clock” approach for measuring the turnover of HIV DNA in resting CD4+ T cells. This approach studies the replacement of wild-type (WT) SIV DNA present in early infection by CTL escape mutant (EM) strains during later infection. Using a strain-specific real time PCR assay, we quantified the relative amounts of WT and EM strains in plasma SIV RNA and cellular SIV DNA. Thus we can track the formation and turnover of SIV DNA in sorted resting CD4+ T cells. We studied serial plasma and PBMC samples from 20 SIV-infected Mane-A*10 positive pigtail macaques that have a signature Gag CTL escape mutation. In animals with low viral load, WT virus laid down early in infection is extremely stable, and the decay of this WT species is very slow, consistent with findings in subjects on anti-retroviral medications. However, during active, high level infection, most SIV DNA in resting cells was turning over rapidly, suggesting a large pool of short-lived DNA produced by recent infection events. Our results suggest that, in order to reduce the formation of a stable population of SIV DNA, it will be important either to intervene very early or intervene during active replication.

## Author Summary

New treatments for HIV have proved very successful at controlling viral replication and preventing the onset of AIDS. However, these treatments must be continued for life, because if they are stopped the virus rapidly ‘rebounds’ to its original levels. The reason for this rebound is the existence of a population of viruses that lie dormant inside cells during treatment, and reactivate as soon as treatment is stopped. This ‘latent virus’ is extremely long-lived under drug therapy conditions, and therefore presents a major barrier to viral eradication. However, very little is known about the survival and reactivation of latently infected cells during ongoing infection, because virus is being formed and destroyed all the time. We have developed a novel ‘escape clock’ approach to measure how long viral DNA lasts in monkeys. We find that, in the setting of low viral load, the lifespan of infected cells is very long, whereas during active infection there is a surprisingly high turnover of viral DNA within resting CD4 T cells. We believe this is due to high level of immune activation when there is a high level of replicating virus. This result may have important implications for the optimal timing of drug treatment.

## Introduction

Treatment of HIV-1 infected individuals with highly active antiretroviral therapy (HAART) can suppress plasma viral RNA levels below the threshold of detection by standard diagnostic assays. However after cessation of even long-term HAART, virus replication is quickly re-established [1]–[4]. A barrier to viral eradication is the presence of viral DNA stably integrated into the chromosomes of resting CD4+ T cells and other long-lived cell populations, since the decay of this viral compartment is very slow [5]–[17].

Several studies suggest that persisting integrated viruses are laid down early in infection [18]–[20]. An indication for this is that HIV strains cultured from resting CD4+ T cells are genetically distinct to concurrent plasma virus [20]. However, it is generally difficult to study the precise kinetics of establishment and turnover of the latent reservoir in most human cohorts as the infecting isolate is usually not known and serial samples available during asymptomatic early infection (1–2 weeks post transmission) are difficult to acquire. Studying macaques experimentally infected with SIV overcomes these barriers. Recent studies show the utility of SIV-infected macaque models for studying long-term integrated viruses [21]–[23].

During HIV and SIV infection one typically sees immune escape at defined cytotoxic T cell lymphocyte (CTL) epitopes. CTL escape mutations (EM) are frequently generated early after the acute infection stage and typically follow predictable patterns of outgrowing wild type (WT) virus. We hypothesised that evidence for the early formation and turnover of SIV DNA may be found by comparing the dynamics of immune escape in cellular viral DNA populations to the dynamics in replicating plasma virus. If replicating WT virus (as indicated by plasma RNA) is only present during acute infection, and is completely replaced in plasma by replicating EM virus during chronic infection, the latent reservoir will be primarily WT if laid down during acute infection, but predominantly EM if laid down (or rapidly turned-over) during chronic infection. In other words, if WT viral DNA is detected in resting CD4+ T cells during chronic infection and remains at similar levels when measured later, this supports low rates of turnover of viral DNA populations during active infection.

We previously developed sensitive real-time PCR assays to quantify EM and WT viruses at a Mane-A*10-restricted SIV Gag CTL epitope (termed KP9) in replicating plasma RNA [24], essentially providing a “viral load” of both WT and EM quasispecies. For this study, we also developed PCR assays to assess WT and EM populations in cellular SIV DNA in FACS-sorted resting CD4+ T cells. After obtaining serial plasma and PBMC samples from Mane-A*10 positive SIV-infected pigtail macaques, we used the observed evolution of WT and EM replicating plasma SIV RNA viruses to model the turnover rate that resulted in the observed relative levels of WT and EM SIV DNA sequences. These analyses suggest that during periods of active high-level infection, the majority of SIV DNA in resting CD4+ T cells is turning over very rapidly. However, at lower levels of infection a substantial proportion of SIV DNA in resting CD4+ T cells is laid down early (when virus is still WT at the CTL epitope) and this WT reservoir persists at stable high levels during chronic infection.

## Results

### Rapid immune escape in plasma virus

**Figure 1:**
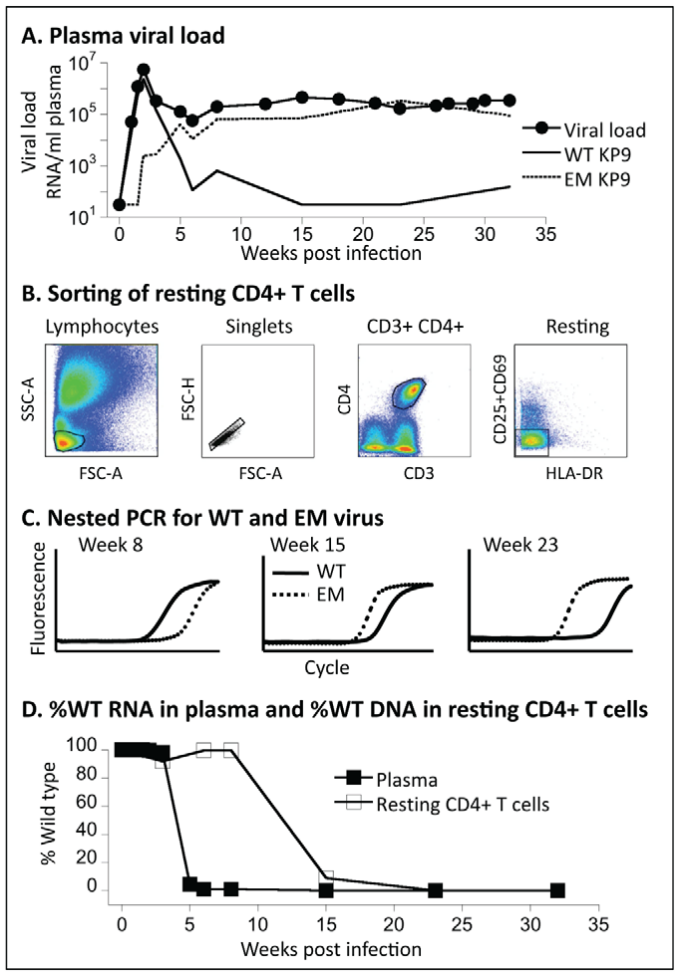
Measuring WT and EM virus in plasma and resting CD4+ T cells. (A) The levels of WT and EM virus in plasma were estimated using a variant-specific real-time PCR assay, shown here for animal 1335. In order to estimate the proportion of WT virus in resting CD4+ T cells, cells were first sorted (B), and then DNA extracted and the levels of WT and EM virus measured using the variant-specific PCR (C). Combining this data, we can plot the fraction of WT virus in the plasma (closed squares) and resting CD4+ T cells (open squares) over time (D).

We first studied 12 Mane-A*10+ animals in a SIV vaccine trial using an influenza virus vector, as this large study provided an extensive bank of plasma and PBMC samples (Table 1). We characterized the frequency of CTL escape mutant and wild-type variants in SIV plasma RNA (Figure 1A). The evolution of EM and WT viruses in plasma was derived by a previously described SIV Gag KP9 qRT-PCR that specifically discriminates the K165R EM virus [24]. After infection with the SIV_mac251_ challenge stock, peak SIV viremia with predominating WT virus was observed ∼2 weeks post infection in all animals. K165R CTL immune escape predictably occurred [24], [25] and EM virus predominated in chronic infection, being selected in preference to WT virus in the majority of animals. Several animals had plasma viremia trajectories in which there was minimal or no detectable EM virus in plasma during acute infection, and complete or near complete replacement of WT virus with EM virus in plasma during chronic infection.

**Table 1:** Macaques studied.

Monkey ID	Challenge Mode	Challenge Dose	Vaccination	ART treatment	Reference
1335	intrarectal	10^4^ TCID50	Flu-KP9*	none	Reece et al Plos One 2010
2374	intrarectal	10^4^ TCID50	Flu-KP9		
25377	intravaginal	2×10^4^ TCID50	Flu-SIV	none	NA
C0933	intravaginal	2×10^4^ TCID50	Flu-SIV		
B0547	intravaginal	2×10^4^ TCID50	Flu-SIV		
B0517	intravaginal	2×10^4^ TCID50	Flu-SIV		
B0526	intravaginal	2×10^4^ TCID50	Flu-SIV		
19341	intravaginal	2×10^4^ TCID50	none		
19351	intravaginal	2×10^4^ TCID50	none		
B0508	intravaginal	2×10^4^ TCID50	none		
C3751	intrarectal	10^4^ TCID50	none		
C5873	intrarectal	10^4^ TCID50	none		
1.3731	intravenous	40 TCID50	15mer peptides spanning all SIV Proteins	Tenofovir (TDF) and Emtricitabine (FTC), week 3 to week 10 after SIV infection	De Rose et al. Plos Path 2008
8014	intravenous	40 TCID50	none		
8020	intravenous	40 TCID50	Gag 15mer peptides		
8244	intravenous	40 TCID50	Gag 15mer peptides		
9021	intravenous	40 TCID50	15mer peptides spanning all SIV Proteins		
9175	intravenous	40 TCID50	15mer peptides spanning all SIV Proteins		
9183	intravenous	40 TCID50	none		
5424	intravenous	40 TCID50	none	none	Smith et al. J Virol. 2004
1.7105	intrarectal	5×10^4^ TCID50	DNA/Fowlpox virus	none	Dale et al. J Virol. 2004

*: Flu-KP9 is recombinant Influenza viruses expressing SIV Gag KP9 CTL epitope; Flu-SIV is recombinant influenza viruses expressing SIV Gag KP9 CTL epitope and 2 SIV Tat CLT epitopes (KSA10 and KVA10).

### Rapid turnover of SIV DNA within resting CD4 T cells during active infection

To assess SIV DNA within resting CD4 T cells, we sorted cells based on their being positive for CD3 and CD4 and negative for HLA-DR and CD69/CD25 (Figure 1B), and then performed a nested PCR with the second round being specific for either WT or the K165R CTL EM virus (Figure 1C). All animals were infected with SIV_mac251_ that is WT at this CTL epitope. The PCR provides relative levels of EM and WT SIV DNA in resting CD4 T cells. We performed assays on FACS-sorted resting CD4 T cells obtained from PBMC samples collected over the course of infection, and compared the ratio of WT and EM virus in plasma with that in SIV DNA in resting cells (Figure 1D).

**Figure 2:**
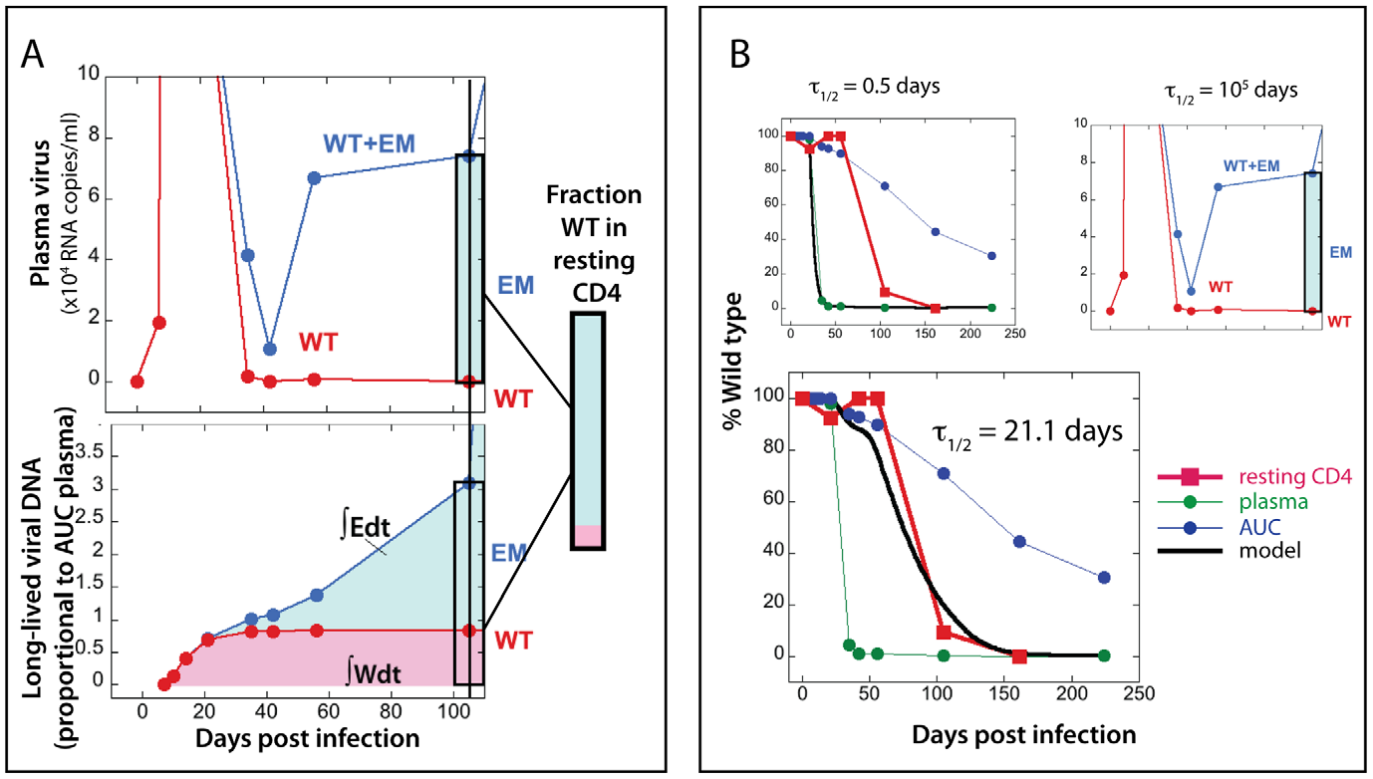
Estimating the half-life of SIV-DNA in resting cells using the ‘escape clock.’ The fitting of the experimental data for animal 1335 is shown to illustrate the modelling approach. Panel A illustrates the approach using a single timepoint late in infection. In the top figure in panel A, the levels of WT (red) and total (WT + EM, blue) virus are plotted over time (on a linear scale). The shaded rectangle indicates the current fraction of WT virus on day 105 post-infection in plasma (pink for WT and light blue for EM). If viral DNA turned over fast (with half-life of half a day or less), both WT and EM would follow the ratio in plasma and we would expect nearly 100% EM SIV DNA in resting CD4+ T cells on day 105. The bottom half of panel A represents the case when viral DNA does not decay (has infinite half-life). In this case, viral DNA accumulates all the time since inoculation, and the amount of each virus type follows the area under the curve (AUC) of WT (∫Wdt) and EM (∫Edt) viral load. The scale of viral DNA is linear in arbitrary units. In this case we would expect a much higher WT fraction in viral DNA in resting cells on day 105 (rectangle). The box in the middle on the right of panel A shows the experimentally measured fraction of WT DNA in resting cells, which is between the two extremes. This implies that the half-life of viral DNA lies between 0.5 days and infinite time. Panel B illustrates the fitting of the optimal half-life of SIV-DNA using the longitudinal data for animal 1335. The green circles are the experimental values of WT virus fraction in plasma over time; the blue circles are the fraction of WT virus estimated from the AUC of viral load. The red squares show the experimentally observed fraction of WT virus SIV DNA in resting CD4+ T cells. The black line illustrates the fraction of WT virus expected from the model (Eq.2) with different values of SIV DNA half-life. The top left figure shows the estimated DNA fraction for half-life of 0.5 days, which follows the experimental plasma fraction closely, but is considerably below the observed DNA fraction. The top right figure shows the DNA fraction estimated from infinite lifetime (area under the curve), which is higher than the observed fraction in the later stage of infection. The larger figure at the bottom shows the estimate using the best-fit lifetime of 21.1 days, which closely follows the observed variation of WT DNA.

These two ratios of WT/EM are the basis of the “escape clock” that we use to estimate the SIV DNA turnover rate in resting cells. The method is outlined in the in Figure 2. Briefly, if SIV DNA turns over quickly (or has a short half-life), then we expect the fraction of WT virus in SIV DNA to closely track the ratio seen in plasma virus, since most of SIV DNA would have been recently formed from plasma virus. If, on the other hand, SIV DNA is extremely stable (or persists indefinitely), then we expect the fraction of WT in SIV DNA to reflect the accumulation of all latently infected cells over the whole previous course of infection. The archived viral DNA of each strain should then be proportional to the ‘area under the curve’ (AUC) of each virus over time. For any SIV DNA ratio in between these extremes, we could estimate the optimal half-life of SIV DNA that best fits the data using the model described in the Methods section.

**Figure 3:**
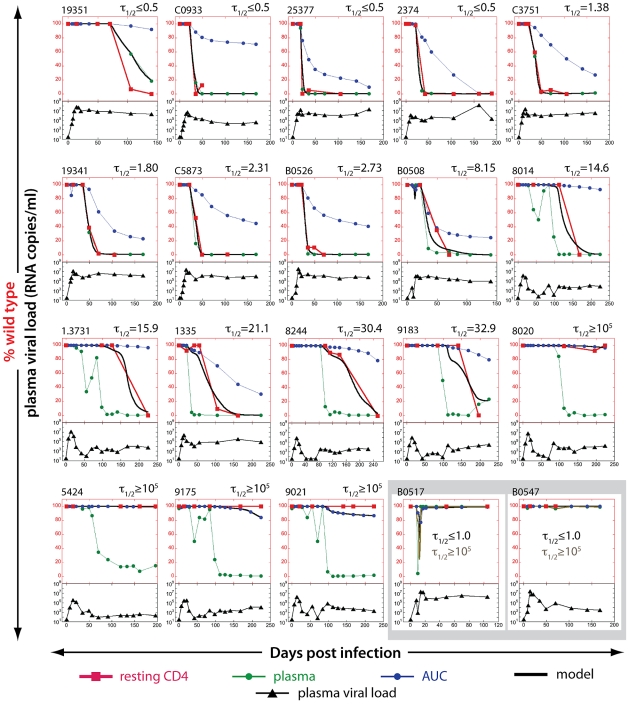
Estimating the half-life of SIV DNA in resting CD4+ T cells. The proportion of WT virus in plasma (green circles), the fraction of WT virus estimated from the area under the curve (AUC) of viral load (blue circles) and the experimentally observed fraction of WT virus SIV DNA in resting CD4+ T cells (red squares) are shown for each animal in the top part of each panel. The black line illustrates the best-fit SIV DNA half-life to the observed fraction of WT virus in resting CD4+ T cells for each animal. Animals are arranged in order of increasing half-life of SIV DNA. The bottom part of each panel (black triangles) represents total plasma viral load. Viral loads are all on the same log_10_ scale, from 10–10^9^. The bottom right two panels (B0517 and B0547) illustrate two animals in which EM appeared only transiently in plasma. In this case, the fraction WT virus is nearly 100% in both plasma and AUC estimates at the time points where DNA was measured, so the ‘escape clock’ fits equally well with a half-life of 1 day or 100,000 days.

In the majority of these animals with active replication (which had high viral loads) we observed that CTL escape in the plasma SIV RNA was closely followed by CTL escape in the SIV DNA from FACS-sorted resting CD4 T cells (first 8 animals in Figure 3). The estimated half-life of SIV DNA was therefore extremely short – of the order of a few days.

**Figure 4:**
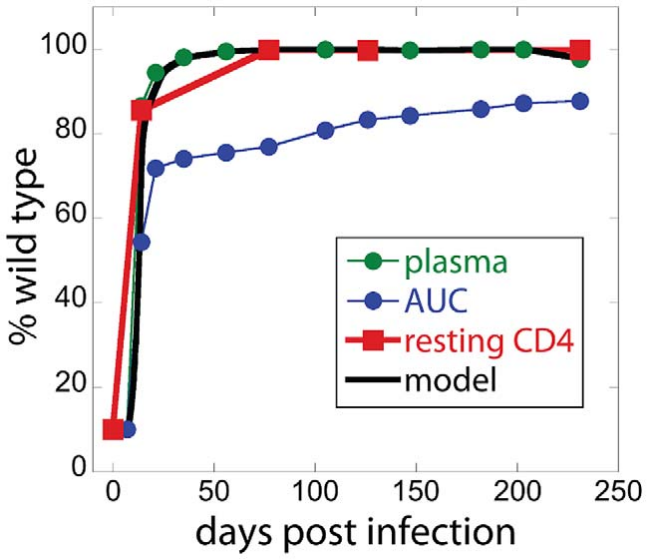
Reversion in animal 1.7105. Mane-A*10 negative animal 1.7105 was challenged with SHIV_mn229_, which is a viral stock containing 10% WT and 90% K165R escape mutation at the Gag KP9 epitope. In the absence of KP9-specific immune response, virus quickly reverts to WT. During reversion, the fraction of WT in resting CD4+ T cells closely follows the fraction in plasma, similarly to the escape pattern in Mane-A*10 positive animals with high chronic viral loads challenged with WT SIV_251_.

To confirm these results we also assessed reversion of the K165R KP9 CTL escape mutation in a Mane-A*10 negative animal infected with the SHIV_mn229_ challenge stock. This challenge stock had previously been passaged in a Mane-A*10+ animal and was composed largely of the K165R escape mutation [26]. In the absence of CTL pressure, we observe plasma SIV RNA rapidly reverting back to wild type, as previously reported [26]. Thus, this provides an ‘escape clock’ where EM instead of WT virus is temporarily expressed, and thus provides an excellent control for our measurements of WT∶EM ratio. Consistent with our findings with WT SIV_mac251_ infection, we found that the cellular SIV DNA in resting CD4 T cells also reverted back to wild type very rapidly in this animal with high levels of viral replication (Figure 4).

### Slow turnover of SIV DNA in animals with low viral loads

The rapid turnover of cellular SIV DNA in resting CD4 T cells that we observed during high level SIV infection above was surprising, given the accepted stability of the latent reservoir in subjects with very low levels of replication on HAART. To investigate the effects of plasma viral turnover on the persistence of SIV DNA, we repeated the study on a cohort of 8 Mane-A*10+ animals from a peptide immunotherapy trial. These animals had undergone ART at week 3, followed by immunotherapy and cessation of ART (week 10), leading to long-term low levels of viral replication in many animals. In these animals, escape was usually observed in the plasma following therapy interruption, leading to the rapid appearance and dominance of EM virus in chronic infection. Thus we were able to study SIV DNA dynamics in resting CD4+ T cells in chronic infection at a time of low viral loads in the absence of therapy. The results of fitting the half-life of WT DNA in animals from both trials are shown in Figure 3, in the order of increasing estimated half-life.

Analysis of the proportion of WT SIV DNA in resting cells from these animals produced a very different picture from that seen in the first cohort. In several animals, the SIV DNA in resting cells remained close to 100% WT, despite EM virus dominating the plasma for prolonged periods. When we estimated the half-life of SIV-DNA in these resting cells using the ‘escape clock’ approach, we found that in 4 animals with very low viral loads the half-life of SIV-DNA in resting cells was >20 years. In several other animals, although some turnover could be measured, the half-lives were extremely long.

**Figure 5:**
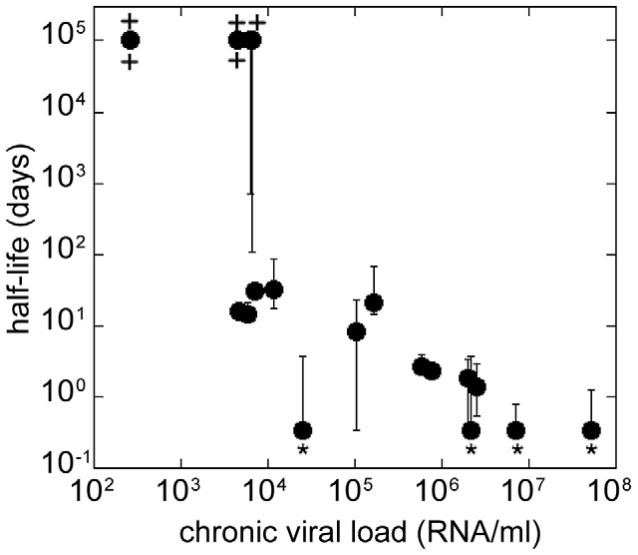
SIV DNA half-life decreases with increasing chronic plasma viral load. The chronic plasma viral load (estimated as the geometric mean viral load from day 100 post-infection) is significantly correlated with the estimated half-life of SIV DNA for each animal (Spearman correlation, r = −0.8358, p<0.0001). The error bars represent confidence intervals (C. I); “*” marks the C. I. limit <0.5 days and “+” marks the C. I. limit >10^5^ days.

We then investigated whether viral load was a correlate of the rate of SIV DNA turnover in sorted resting CD4+ T cells. We observed a significant correlation between viral load and estimated SIV DNA half-life (Figure 5), suggesting that the high levels of infection and CD4+ T cell activation may play a role in determining SIV DNA turnover.

## Discussion

Current HAART regimes suppress plasma HIV RNA to very low levels, but cessation of HAART results in a brisk rebound of plasma virus [1]–[4]. Cellular compartments containing viral DNA provide a stable long-term reservoir for the virus [5]–[17]. However, the dynamics of establishment and turnover of this reservoir are not well understood. In particular, the majority of studies of HIV latency have focused on HIV DNA turnover under therapy, when viral replication and CD4+ T cell activation and turnover are greatly suppressed. But is HIV DNA persistence the same during active infection?

Our approach using a qRT-PCR to track the evolution of CTL escape mutants allowed us to compare EM and WT virus in plasma RNA and cellular DNA in a cohort of Mane-A*10 positive SIV-infected pigtail macaques. By analysing SIV DNA in purified resting CD4 T cells and comparing with plasma virus, we show that WT SIV DNA can persist in some animals for many months, even where there is an absence of WT viral RNA in replicating plasma virus. Thus, the WT SIV DNA species that are laid down early during infection can persist into late infection, and turnover of this viral compartment is very slow. Importantly however, this long half-life of WT DNA was only seen in animals with low viral loads. Animals with a high viral load showed a very rapid turnover of WT SIV DNA in resting CD4+ T cells. Indeed, we observed a highly significant correlation between the average viral load in chronic infection and the estimated half-life of SIV DNA. This correlation suggests that viral load is an important factor driving SIV DNA turnover in resting CD4 T cells during active infection.

Our observation of the long half-life of SIV DNA associated with low levels of plasma virus is consistent with the previous studies of HIV DNA persistence under drug therapy, where viral loads are even lower than those observed here [14]. What is less clear from our study is why we see such a rapid SIV DNA turnover in resting cells during active infection. A number of mechanisms are possible. Firstly, it seems possible that CD4+ T cells simply don't get a chance to stay in the resting state long enough to maintain a stable integrated pool, since SIV DNA is continuously driven to productive infection because of host cell activation. The half-life that we are estimating here is then half-life spent in the “resting” pool, i.e. the time during which infected cells express CD3 and CD4 and are negative for HLA-DR, CD69 and CD25. When they activate, they are no longer sorted as resting, and are lost from the pool in the same way as if they died. One limitation to our conclusions about the latent infection is that they apply only to CD69^−^CD25^−^HLA-DR^−^ infected CD4+ T cells in blood, which may not truly represent the latent pool, but may be a heterogeneous population containing truly latently infected cells as a small subset. Thus, although the observed average turnover of HIV DNA in these resting CD4+ T cells is sometimes extremely fast, we cannot exclude that there might be minor populations of cells harbouring much longer-lived DNA, or that indeed long-lived DNA might not be harboured at some other anatomical site. Although we also found very few effector memory or dividing cells within the sorted resting CD4 T cell population, future studies could sort even more highly refined resting CD4 T cell populations or investigate other anatomical sites to evaluate this further.

A second possibility is that the observed SIV DNA represents a mix of long-lived, integrated SIV DNA, and of short lived reverse transcription products that represent dead-ends for the virus. At low viral loads, the level of short-lived reverse transcription products is very low, as there is little virus present in plasma to produce new infections. Thus, the SIV DNA observed comes predominantly from the long-lived integrated pool, and we observe the slow SIV DNA turnover characteristic of this compartment. However, in animals with a high viral load, we may see a high level of recent infection and of short-lived reverse-transcripts. If viral loads are high enough, this pool of recent reverse transcripts is the dominant population we see, overwhelming the long-lived WT DNA, and leading to an apparent close tracking of the viral DNA in resting cells with the plasma DNA. This mechanism also predicts that the long-lived WT DNA pool always persists at the same level, but is numerically overwhelmed by the large number of copies of short-lived EM DNA when plasma viral load is high.

In our model we did not consider a possibility that the half-life of infected resting cells depends on viral strain, because we assumed that they would not express viral epitopes and would not be recognized while resting. In addition, the fraction of WT DNA in resting cells is in most cases higher than in plasma, which is not supportive of preferential killing of resting cells with WT DNA. However, it is in principle possible that WT-infected resting cells are preferentially killed during periods of fast turnover, when the WT fraction is very low and approaches that in plasma. We note though that if preferential killing of WT infected resting CD4+ T cells were driving the rapid turnover of latency, we might expect that the turnover would be correlated with CTL number.

Specifically, if this were the mechanism driving the turnover, we would expect that in animals with good CTL control (low viral loads) we should see faster turnover of HIV-DNA. However, we observed the opposite relationship. Moreover, when we analysed the correlation between the number of tetramer positive cells and HIV-DNA turnover, we found that both in early (before the appearance of EM in plasma) and in chronic infection the average number of tetramer positive cells was positively correlated with the estimated half-life of resting infected cells. This is in agreement with our interpretation that better immune control leads to less reactivation of latently infected cells.

**Figure 6:**
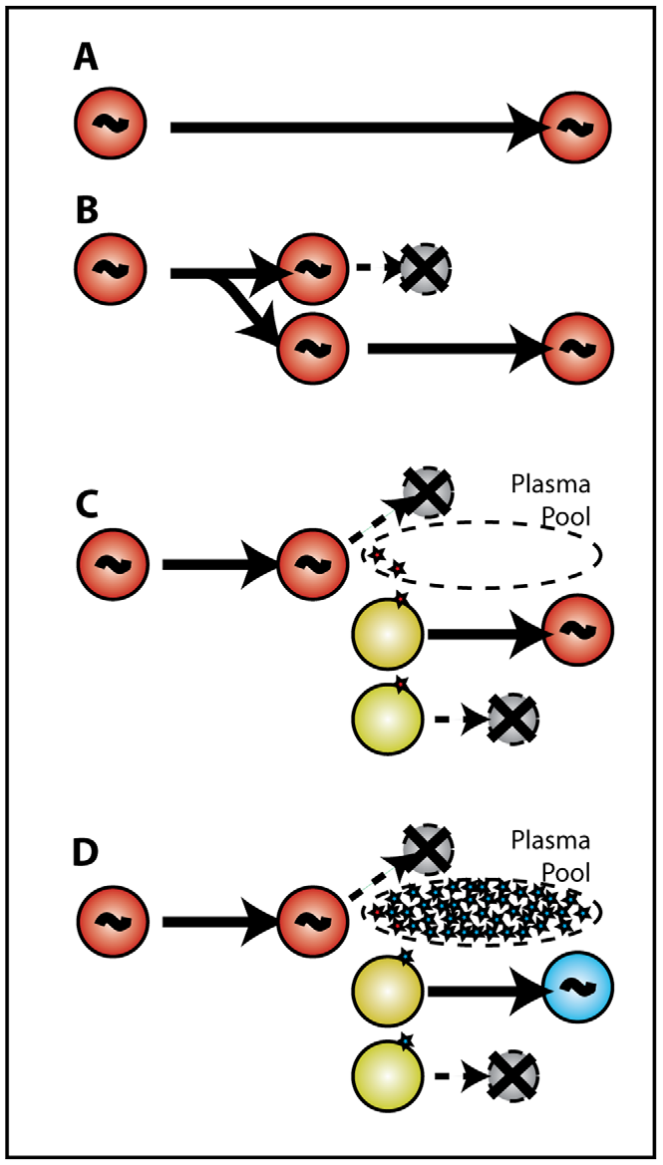
SIV DNA persistence does not require reinfection. Several mechanisms have been postulated for the persistence of HIV DNA under prolonged therapy. (A) Infected cells harbouring HIV DNA may be long-lived, or (B) may undergo homeostatic replication, in which the daughter cell carries the HIV DNA. Panel C illustrates one possible mechanism of viral persistence under therapy, where latent cells become reactivated, shed virus and die, leading to the infection of a new generation of cells. However, during active infection (D), activation of latent WT infected cells (red) leads to production of small amounts of WT virus (red) that is effectively diluted by the abundance of EM virus in plasma (blue). Thus, reinfection is predominantly with EM virus, and the WT is effectively removed by activation and replication. The long-term persistence of WT SIV-DNA despite EM dominance in plasma, at levels at or above the AUC estimate (in animals 9021, 9175, 5424, 8020) make reinfection a very unlikely contributor to SIV DNA persistence.

Our analyses are in agreement with early studies in humans suggesting that latent reservoirs in resting CD4+ T cells are laid down earlier in infection and are extremely long-lived [20], [27]. There are a number of possible mechanisms by which long-lived SIV/HIV DNA may persist in cells (illustrated in Figure 6). Firstly, the individual cells bearing HIV DNA may be extremely long-lived. Secondly, these cells may turn over through homeostatic replication, with a balance of cell replication and death leading to a stable number of HIV DNA copies. Finally, it has been suggested that HIV persistence may be maintained by low levels of viral reactivation, replication, and reinfection of new cells, leading to a stable level of HIV DNA copies. This latter mechanism seems unlikely at low plasma viral loads given our results. That is, if WT DNA persistence involved reactivation, viral production into the plasma, and reinfection of new cells, then we should be able to estimate the proportion of new infections due to WT virus, by the ratio of WT∶ EM virus in the plasma. However, since in most cases we don't observe any WT virus in the plasma in chronic infection, it could at best make only a very trivial contribution to any reinfection, and could not maintain WT DNA levels at or above the AUC levels via this mechanism.

There are limitations to our data that suggest further studies. Discriminating integrated from non-integrated forms of SIV DNA was not feasible in the small numbers of sorted resting CD4 T cells without further optimising the assay. Several assays have been designed to measure the more abundant non-integrated forms of HIV/SIV such as 1 LTR and 2-LTR circular forms, and these could be used in the future to determine the level of contaminating non-integrated SIV [28]–[31]. No widely accepted method exists to measure the “true” latent reservoir and our studies of resting CD4 T cells are only an approximation of this as yet undefined cell population. PCR-based assays have the disadvantage in that much of the cellular HIV-1 DNA may be from replication-deficient virus, although for viruses to contribute to the latent reservoir they must be replication-competent [20], [32]. However, our analyses focus only on a single nucleotide change in the KP9 CTL epitope. This change (alone) is replication competent and it seems unlikely that additional lethal mutations would accumulate more commonly in either WT or the K165R EM species.

Our results provide a method for direct quantification of HIV DNA turnover during active infection, and show for the first time that SIV DNA turnover in resting CD4+ T cells is strongly correlated with viral load during chronic infection. The rapid turnover of SIV DNA in animals with high viral load suggests the resting pool of CD4+ T cells and the pool of SIV DNA may be much more dynamic than previously thought during active infection. However, the persistence of WT SIV DNA laid down in early infection in animals with low chronic viral loads indicates the importance of the earliest stages of infection in establishing the latent pool of HIV DNA. Taken together, our study highlights the importance of early viral control in preventing the establishment of persistent HIV infection.

## Materials and Methods

### Ethics statement

Experiments on pigtail macaques (*Macaca nemestrina*) were approved by CSIRO livestock industries Animal Ethics Committees (approval number 1315) and cared for in accordance with Australian National Health and Medical Research Council guidelines.

### Animals

We studied serial PBMC and plasma samples from 20 pigtail macaques involved in several SIV infection studies [33], [34]. Five macaques received no SIV vaccinations and were infected with SIV_mac251_ (wild type at KP9) [35]. Two macaques received influenza viruses expressing KP9 and were infected with SIV_mac251_ [34]. Five macaques received influenza viruses expressing KP9, KSA10 and KVA10. Eight Mane-A*10 positive pigtail macaques were enrolled in a therapeutic peptide-based vaccine trial [33]. The outline of the therapeutic study was as follows: pigtail macaques were infected intravenously with SIV_mac251_ at week 0 and received ART (tenofovir and emtricitibine) from weeks 3 to 10 post infection and then withdrawn. Either no treatment (controls) or OPAL treatment (overlapping 15mer Gag peptides only or peptides from all 9 SIV proteins) was given at weeks 4, 6, 8 and 10 after infection. PBMC and plasma samples were collected at regular time points on all animals. The animals and their treatment are summarized in Table 1.

### Analyses of WT and EM virus in plasma RNA

To quantify virus levels of WT or EM quasispecies at the KP9 epitope we employed a discriminatory real-time PCR assay as described [24], [36]. Briefly, the assay uses a forward primer specific for either wild-type sequence or specific for the nucleotide mutation encoding the dominant K165R KP9 escape mutant. At each timepoint after infection 10 µl of plasma RNA was reverse-transcribed and then amplified by qRT-PCR using either WT or EM forward primers. A common reverse primer and FAM-labelled DNA probe were also added for quantification against the appropriate SIV Gag epitope RNA standards using an Eppendorf Realplex^4^ cycler. Analysis was performed using Eppendorf Realplex software. Baselines were set 2 cycles earlier than real reported fluorescence and threshold value was determined by setting threshold bar within the linear data phase. Samples amplifying after 40 cycles were regarded as negative, and corresponded to <1.5-Log_10_ SIV RNA copies/ml of plasma. Plasma viral cDNA was also subjected to Sanger-based sequencing as previously described [37] to confirm the EM quasispecies contained the K165R mutation detected in the qRT-PCR assay.

### Sorting resting CD4 T cells

We studied HLA-DR-CD69−CD25− CD4+CD3+ T lymphocytes as resting CD4 T cells as these cells are commonly studied as a model for resting CD4 T cells [32], [38]–[41]. Frozen PBMC (approximately 5×10^6^) were thawed at 37°C in RF10, centrifuged at 300 g and resuspended in 500 ul of PBS containing 2 mM EDTA. 0.5 µl live/dead (Near Infra Red –IR (APC-Cy7) viability stain/tube was added and tubes were incubated for 60 minutes on ice in the dark. Cells were washed for 5 min at 500 g, the supernatant removed and surface stained with an antibody cocktail of CD69-APC (clone L78), CD3-PE (clone SP34-2), CD4-FITC (clone L200), CD25-APC (clone BC96) and HLA-DR- PerCP Cy5.5 (clone L243) on ice in the dark for 60 minutes to avoid CD4 T cell activation. PBMC were washed in PBS containing 0.5% BSA and 2 mM EDTA, fixed in 0.1% formaldehyde and passed through filtered facs tube prior to being sorted on the FACSAria. Live resting CD4+ T cells were positive for CD3 and CD4 and negative for HLA-DR and CD69/CD25. To validate whether HLA-DR-CD69−CD25− CD4+CD3+ T lymphocytes studied were truly resting using other markers we also studied CD28 and CD29 memory markers and the cell turnover marker Ki67 in a subset of the studied animals. The activated cells were highly enriched for CD28−95+ effector memory cells. A mean of only 0.91% of the resting cells were of the effector memory phenotype (p<0.001). The activated cells were also highly enriched for Ki67 staining (p = 0.0028). A mean of only 1.64% of the resting cells were Ki67+.

### Nested real-time PCR to assess WT and EM SIV DNA

DNA from sorted cells was extracted using the Qiagen mini DNA. A nested KP9-specific qRT-PCR was performed that consisted of a first round Gag specific PCR followed by a second round discriminatory KP9 qRT-PCR. The first round PCR utilized 400 nM of the Gag forward primer (5′- CAAGTAGACCAACAGCACCATCTAGCGGCAG-3′) and reverse primer (5′- CTTGTTGTGGAGCTGGTTGTGGGTGCTGCAAGTC). Amplification was performed using 2 U Expand High Fidelity polymerase, 200 nM dNTPs and 250 mM MgCl_2_ per reaction. Amplification consisted of 94°C for 2 minutes followed by 22 cycles of 94°C for 15 seconds, 68°C for 30 seconds and 72°C for 45 seconds, with a final extension of 72°C for 7 minutes. The second round dKP9 qRT-PCR has previously been described for the analysis of WT and EM virus in plasma RNA, was performed [24], [36]. The second round product was serially diluted 1/100 to 1/2000 to ensure that the second round qRT-PCR did not contain saturating amounts of DNA. Sanger based sequencing confirmed the ratios of WT∶EM virus observed in the real-time PCR reaction (not shown).

### Modeling SIV DNA turnover

We start from a simple model describing WT and EM infection in resting CD4+ T cells:
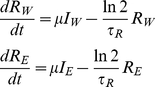
(1)In this model, cells infected with WT and EM (*I_W_* and *I_E_* respectively) are becoming resting (*R_W_* and *R_E_* for resting cells infected with WT or EM respectively) at a fixed rate *μ* and have a half-life of *τ_R_*. Half-life can describe the loss of resting cells either to death or to activation. We do not have access to *I_W_* and *I_E_* from experiment, but we assume that free virus is turning over much faster than productively infected cells [42], so that plasma virus to a good approximation reflects the corresponding productively infected cell level. This proportionality holds irrespective of the cause of viral load variation – be it because of the variation in target cell numbers, immune response or drug therapy. It allows us to replace *μI_W_* and *μI_E_* in Eq.1 with *fW* and *fE*, where *W* and *E* are the plasma WT and EM viral loads, and *f* is a constant different from *μ*.

We then use this model to estimate the half-life of viral DNA in resting infected cells using the measured WT and EM viral loads and the fraction of WT DNA in resting cells *b_W_* = *R_W_*/(*R_W_*+*R_E_*). For this purpose we rewrite the system Eq.1 (with *W* and *E* replacing *I_W_* and *I_E_*) in terms of the WT fraction *b_W_* and a variable Λ representing total number of infected resting cells scaled by constant *f*, Λ = (*R_W_*+*R_E_*)/*f*:
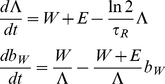
(2)The system Eq.2 has only one fitting parameter, the half-life of viral DNA *τ_R_*. We find the best fit of *τ_R_* for each animal by solving Eq.2 with initial conditions Λ(*t* = 0) = 1 and *b_W_*(*t* = 0) = 1 (because inoculating SIV_mac251_ is 100% WT) and choosing a value of *δ* = ln2/*τ_R_* between 0 and 2 such that it minimizes the deviations from measured points of WT DNA fraction, using measured values of WT and EM viral load with exponential interpolation between time points for the variables *W* and *E*. Because the fitted values of the WT fraction *b_W_* must lie between 0 and 1, the best-fit value of *δ* has to minimize the expression [43]:
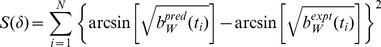
(3)where *t_i_* are the time points when the measurements were taken, *b_W_^expt^* are the measured values of the WT fraction at this point, and *b_W_^pred^* are the values predicted by the model. The arcsin-square root transformation of the deviations was used to normalize the error distribution.

Given the initial conditions, the solution Λ(*t*), *b_W_*(*t*) of the system Eq.2 for each value of *τ_R_* is unique. Therefore it is sufficient to have one experimental WT fraction different from 1 or 0 to completely define the trajectory of WT fraction in time. We have such points for all animals. Even when a fraction looks like 1 or 0, it often deviates a little from these numbers. The largest possible value of τ_R_ that could be obtained from our model is infinity (which we report as >10^5^ because *δ* = 10^−5^ is the lowest parameter value used that was greater than zero), and the lowest possible value is 0.35 days (if this is the best fit, we report it as <0.5 days).

The confidence intervals for each animal in Figure 5 are obtained by bootstrapping, using the errors transformed by arcsin-square root. The initial point *b_W_*(0) = 1 was not used in bootstrapping. Because the total number of measurements for each animal was small, we used the whole set of error permutations to determine the bounds of the confidence intervals.

Our model assumes that the constant *f* is the same for cells infected with WT and EM. However, this constant may be higher or lower in one of the strains, depending on its propensity *μ* to generate latently infected cells, fitness cost of mutation and type of immune response. We have therefore repeated the process by simultaneously fitting the ratio *f_E_*/*f_W_* and *τ_R_* and found that this did not change the range of observed half-lives or the correlation of half-lives with chronic viral load. The details of this analysis are shown in the Text S1 in the online Supplementary material.

It should be noted that *R_W_* and *R_E_*, which we regarded as infected resting cells in Eq.1 and Eq.2, can also be interpreted as the WT or EM DNA content in resting cells (just as *I_W_* and *I_E_* can represent viral DNA in productively infected cells), In this case, the half-life of *τ_R_* describes the loss of viral DNA due to cell death, degradation or resting cells becoming activated.

## Supporting Information

Text S1 Estimate of half-life of resting infected CD4+ T cells when *f_W_*≠*f_E_*. We derive the extended model where the rates of infected cells becoming latent differ between cells infected with WT and EM and we show that the negative correlation between half-life of latently infected cells and chronic viral load is preserved in the 2-parameter fit.
(PDF)

## References

[1] Fischer M, Hafner R, Schneider C, Trkola A, Joos B (2003). HIV RNA in plasma rebounds within days during structured treatment interruptions.. AIDS.

[2] Douek DC, Brenchley JM, Betts MR, Ambrozak DR, Hill BJ (2002). HIV preferentially infects HIV-specific CD4+ T cells.. Nature.

[3] Fagard C, Oxenius A, Gunthard H, Garcia F, Le Braz M (2003). A prospective trial of structured treatment interruptions in human immunodeficiency virus infection.. Arch Intern Med.

[4] Ruiz L, Carcelain G, Martinez-Picado J, Frost S, Marfil S (2001). HIV dynamics and T-cell immunity after three structured treatment interruptions in chronic HIV-1 infection.. AIDS.

[5] Chun TW, Davey RT, Engel D, Lane HC, Fauci AS (1999). Re-emergence of HIV after stopping therapy.. Nature.

[6] Chun TW, Finzi D, Margolick J, Chadwick K, Schwartz D (1995). In vivo fate of HIV-1-infected T cells: quantitative analysis of the transition to stable latency.. Nat Med.

[7] Chun TW, Carruth L, Finzi D, Shen X, DiGiuseppe JA (1997). Quantification of latent tissue reservoirs and total body viral load in HIV-1 infection.. Nature.

[8] Finzi D, Hermankova M, Pierson T, Carruth LM, Buck C (1997). Identification of a reservoir for HIV-1 in patients on highly active antiretroviral therapy.. Science.

[9] Wong JK, Hezareh M, Gunthard HF, Havlir DV, Ignacio CC (1997). Recovery of replication-competent HIV despite prolonged suppression of plasma viremia.. Science.

[10] Chun TW, Stuyver L, Mizell SB, Ehler LA, Mican JA (1997). Presence of an inducible HIV-1 latent reservoir during highly active antiretroviral therapy.. Proc Natl Acad Sci U S A.

[11] Igarashi T, Brown CR, Endo Y, Buckler-White A, Plishka R (2001). Macrophage are the principal reservoir and sustain high virus loads in rhesus macaques after the depletion of CD4+ T cells by a highly pathogenic simian immunodeficiency virus/HIV type 1 chimera (SHIV): Implications for HIV-1 infections of humans.. Proc Natl Acad Sci U S A.

[12] Sonza S, Maerz A, Deacon N, Meanger J, Mills J (1996). Human immunodeficiency virus type 1 replication is blocked prior to reverse transcription and integration in freshly isolated peripheral blood monocytes.. J Virol.

[13] Siliciano JD, Kajdas J, Finzi D, Quinn TC, Chadwick K (2003). Long-term follow-up studies confirm the stability of the latent reservoir for HIV-1 in resting CD4+ T cells.. Nat Med.

[14] Chun TW, Justement JS, Moir S, Hallahan CW, Maenza J (2007). Decay of the HIV reservoir in patients receiving antiretroviral therapy for extended periods: implications for eradication of virus.. J Infect Dis.

[15] Pierson T, McArthur J, Siliciano RF (2000). Reservoirs for HIV-1: mechanisms for viral persistence in the presence of antiviral immune responses and antiretroviral therapy.. Annu Rev Immunol.

[16] Strain MC, Gunthard HF, Havlir DV, Ignacio CC, Smith DM (2003). Heterogeneous clearance rates of long-lived lymphocytes infected with HIV: intrinsic stability predicts lifelong persistence.. Proc Natl Acad Sci U S A.

[17] Seshamma T, Bagasra O, Trono D, Baltimore D, Pomerantz RJ (1992). Blocked early-stage latency in the peripheral blood cells of certain individuals infected with human immunodeficiency virus type 1.. Proc Natl Acad Sci U S A.

[18] Droge W, Hack V, Breitkreutz R, Holm E, Shubinsky G (1998). Role of cysteine and glutathione in signal transduction, immunopathology and cachexia.. Biofactors.

[19] Ruff CT, Ray SC, Kwon P, Zinn R, Pendleton A (2002). Persistence of wild-type virus and lack of temporal structure in the latent reservoir for human immunodeficiency virus type 1 in pediatric patients with extensive antiretroviral exposure.. J Virol.

[20] Monie D, Simmons RP, Nettles RE, Kieffer TL, Zhou Y (2005). A novel assay allows genotyping of the latent reservoir for human immunodeficiency virus type 1 in the resting CD4+ T cells of viremic patients.. J Virol.

[21] Dinoso JB, Rabi SA, Blankson JN, Gama L, Mankowski JL (2009). A simian immunodeficiency virus-infected macaque model to study viral reservoirs that persist during highly active antiretroviral therapy.. J Virol.

[22] Shen A, Zink MC, Mankowski JL, Chadwick K, Margolick JB (2003). Resting CD4+ T lymphocytes but not thymocytes provide a latent viral reservoir in a simian immunodeficiency virus-Macaca nemestrina model of human immunodeficiency virus type 1-infected patients on highly active antiretroviral therapy.. J Virol.

[23] Nishimura Y, Sadjadpour R, Mattapallil JJ, Igarashi T, Lee W (2009). High frequencies of resting CD4+ T cells containing integrated viral DNA are found in rhesus macaques during acute lentivirus infections.. Proc Natl Acad Sci U S A.

[24] Loh L, Petravic J, Batten CJ, Davenport MP, Kent SJ (2008). Vaccination and timing influence SIV immune escape viral dynamics in vivo.. PLoS Pathog.

[25] Smith MZ, Fernandez CS, Chung A, Dale CJ, De Rose R (2005). The pigtail macaque MHC class I allele Mane-A*10 presents an immundominant SIV Gag epitope: identification, tetramer development and implications of immune escape and reversion.. J Med Primatol.

[26] Fernandez CS, Stratov I, De Rose R, Walsh K, Dale CJ (2005). Rapid viral escape at an immunodominant simian-human immunodeficiency virus cytotoxic T-lymphocyte epitope exacts a dramatic fitness cost.. J Virol.

[27] Chun TW, Engel D, Berrey MM, Shea T, Corey L (1998). Early establishment of a pool of latently infected, resting CD4(+) T cells during primary HIV-1 infection.. Proc Natl Acad Sci U S A.

[28] Kumar R, Vandegraaff N, Mundy L, Burrell CJ, Li P (2002). Evaluation of PCR-based methods for the quantitation of integrated HIV-1 DNA.. J Virol Methods.

[29] Butler SL, Hansen MS, Bushman FD (2001). A quantitative assay for HIV DNA integration in vivo.. Nat Med.

[30] Pierson TC, Kieffer TL, Ruff CT, Buck C, Gange SJ (2002). Intrinsic stability of episomal circles formed during human immunodeficiency virus type 1 replication.. J Virol.

[31] Furtado MR, Callaway DS, Phair JP, Kunstman KJ, Stanton JL (1999). Persistence of HIV-1 transcription in peripheral-blood mononuclear cells in patients receiving potent antiretroviral therapy.. N Engl J Med.

[32] Han Y, Wind-Rotolo M, Yang HC, Siliciano JD, Siliciano RF (2007). Experimental approaches to the study of HIV-1 latency.. Nat Rev Microbiol.

[33] De Rose R, Fernandez CS, Smith MZ, Batten CJ, Alcantara S (2008). Control of viremia and prevention of AIDS following immunotherapy of SIV-infected macaques with peptide-pulsed blood.. PLoS Pathog.

[34] Sexton A, De Rose R, Reece JC, Alcantara S, Loh L (2009). Evaluation of recombinant influenza virus-simian immunodeficiency virus vaccines in macaques.. J Virol.

[35] Smith MZ, Dale CJ, De Rose R, Stratov I, Fernandez CS (2005). Analysis of pigtail macaque major histocompatibility complex class I molecules presenting immunodominant simian immunodeficiency virus epitopes.. J Virol.

[36] Loh L, Kent SJ (2008). Quantification of simian immunodeficiency virus cytotoxic T lymphocyte escape mutant viruses.. AIDS Res Hum Retroviruses.

[37] Loh L, Batten CJ, Petravic J, Davenport MP, Kent SJ (2007). In vivo fitness costs of different Gag CD8 T-cell escape mutant simian-human immunodeficiency viruses for macaques.. J Virol.

[38] Tran TA, de Goer de Herve MG, Hendel-Chavez H, Dembele B, Le Nevot E (2008). Resting regulatory CD4 T cells: a site of HIV persistence in patients on long-term effective antiretroviral therapy.. PLoS One.

[39] Gondois-Rey F, Biancotto A, Pion M, Chenine AL, Gluschankof P (2001). Production of HIV-1 by resting memory T lymphocytes.. AIDS.

[40] Sankatsing SU, van Praag RM, van Rij RP, Rientsma R, Jurriaans S (2003). Dynamics of the pool of infected resting CD4 HLA-DR- T lymphocytes in patients who started a triple class five-drug antiretroviral regimen during primary HIV-1 infection.. Antivir Ther.

[41] Wind-Rotolo M, Durand C, Cranmer L, Reid A, Martinson N (2009). Identification of nevirapine-resistant HIV-1 in the latent reservoir after single-dose nevirapine to prevent mother-to-child transmission of HIV-1.. J infect Dis.

[42] Perelson AS, Neumann AU, Markowitz M, Leonard JM, Ho DD (1996). HIV-1 dynamics in vivo: virion clearance rate, infected cell life-span, and viral generation time.. Science.

[43] Mandl JN, Regoes RR, Garber DA, Feinberg MB (2007). Estimating the effectiveness of simian immunodeficiency virus-specific CD8+ T cells from the dynamics of viral immune escape.. J Virol.

