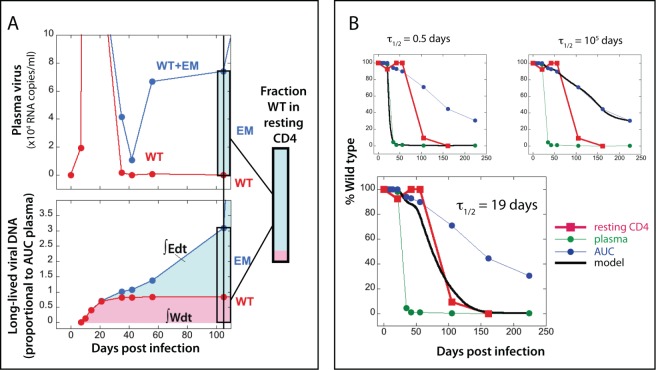# Correction: An “Escape Clock” for Estimating the Turnover of SIV DNA in Resting CD4+ T Cells

**DOI:** 10.1371/annotation/7556ed6f-2d66-48a9-be38-4aefe4400393

**Published:** 2013-09-26

**Authors:** Jeanette Reece, Janka Petravic, Mehala Balamurali, Liyen Loh, Shayarana Gooneratne, Rob De Rose, Stephen J. Kent, Miles P. Davenport

In Figure 2, the top right hand panel in part B is a duplicate of the top panel in part A. The figure legend is accurate.

The correct figure can be downloaded from the following link:

**Figure:**