# Supplementary material for: An “Escape Clock” for Estimating the Turnover of SIV DNA in Resting CD4+ T Cells
Source: PLoS Pathog. 2012 Apr 5;8(4):e1002615. doi: 10.1371/journal.ppat.1002615 (PMC3320584; doi:10.1371/journal.ppat.1002615)
Supplement: Text S1 — Estimate of half-life of resting infected CD4+ T cells when fW≠fE. We derive the extended model where the rates of infected cells becoming latent differ between cells infected with WT and EM and we show that the negative correlation between half-life of latently infected cells and chronic viral load is preserved in the 2-parameter fit. (PDF) [file ppat.1002615.s001.pdf]

SUPPLEMENTARY MATERIAL:

ESTIMATE OF HALF-LIFE OF RESTING INFECTED CD4+ T CELLS WHEN $f_W \neq f_E$

The relationship between the fraction of WT-infected resting cells and viral load may be affected by our assumption that WT- and EM-infected resting cells are created by the virus at the same rate *f*. We outline below that, when we allow this rate to differ for WT and EM in our model, it does not change the observed correlation:

In the extended model we assume:

(i) That the number of new resting cells becoming infected at any time is proportional to the current number of productively infected cells. Generally:

$$\frac{dR_W}{dt} = \mu_W I_W - \frac{\ln 2}{\tau_R} R_W$$

$$\frac{dR_E}{dt} = \mu_E I_E - \frac{\ln 2}{\tau_R} R_E \qquad \text{(S1)}$$

There is no reason to assume that the constants μ_W and μ_E are equal.

(ii) That the number of productively infected cells is proportional to viral load at any time. Again, this is a plausible starting hypothesis, also reproduced in the standard model of viral dynamics. Generally, we have:

$$I_W = \kappa_W W$$

$$I_E = \kappa_E E \qquad \text{(S2)}$$

In the standard model, the constants κ_W and κ_E would be associated with virus production p_W, p_E and clearance c_W, c_E:

$$\kappa_W \approx c_W / p_W$$

$$\kappa_E \approx c_E / p_E \qquad \text{(S3)}$$

We combine these two assumptions to express the equations for resting infected cells in terms of viral load instead of productively infected cells:

$$\frac{dR_W}{dt} = \mu_W \kappa_W W - \frac{\ln 2}{\tau_R} R_W$$

$$\frac{dR_E}{dt} = \mu_E \kappa_E E - \frac{\ln 2}{\tau_R} R_E \qquad \text{(S4)}$$

and we call the combined constants:

$$f_W = \mu_W \kappa_W$$

$$f_E = \mu_E \kappa_E \qquad \text{(S5)}$$

In standard model, we would have:

$$f_W \approx \mu_W c_W / p_W$$

$$f_E \approx \mu_E c_E / p_E \qquad \text{(S6)}$$

Now, escape mutations usually carry a fitness cost that reduces their replicative capacity. Assuming that we can describe the process in terms of the standard model, this would mean that the product of infectivity and viral production is lower in the escape mutant.

However, because of the immune pressure on the wild-type virus, escape mutant ultimately prevails. This can be achieved in several ways depending on the effect of the escape mutation and the type of immune response. Each possibility has a different impact on the relationship between the constants κ_W and κ_E. In particular:

(i) If the immune response is cytolytic, then the lifespan of WT-infected productive cells is reduced, and the replicative capacity of EM stays lower than the replicative capacity of WT. In this case,
  (a) if replicative capacity of EM is lower because its infectivity is lower, but production rate is not, then $\kappa_W \approx \kappa_E$;
  (b) if fitness cost reduces EM production rate, then $\kappa_W < \kappa_E$.

(ii) If the main type of immune response is noncytolytic, it may suppress infectivity or production rate of WT, so that its replicative capacity falls below EM.
  (a) If immune response reduces infectivity of WT and fitness cost reduces infectivity of EM, then $\kappa_W \approx \kappa_E$;
  (b) If immune response reduces infectivity of WT and fitness cost reduces production rate of EM, then $\kappa_W < \kappa_E$;
  (c) If immune response reduces production of WT and fitness cost reduces infectivity of EM, then $\kappa_W > \kappa_E$;
  (d) If immune response reduces production of WT and fitness cost reduces production of EM, then $\kappa_W > \kappa_E$ because there is escape.

In addition, the propensity of each strain to create infected resting cells can in principle differ in any direction: $\mu_W < \mu_E$, $\mu_W \approx \mu_E$ or $\mu_W < \mu_E$.

Even with a simple interpretation of the standard model, it is evident that the presence of fitness cost and noncytolytic immune response does not indicate any specific relationship between f_W and f_E. For a rough estimate of resting cell lifespan we initially used the simplest assumption – that they were equal: $f_W = f_E = f$, in the spirit of Occam's razor.

However, the difference between WT content in plasma and in resting cells may be the result of a difference between f_W and f_E. We therefore investigated the possible effect of this difference and show below that our estimates of the range of half-lives and our finding of the negative correlation between chronic viral load and half-life of resting infected cells are not affected by a difference between f_W and f_E.

If $f_E/f_W = a$, then Eq.(1) in the manuscript would change to:

$$\begin{aligned} \frac{dR_W}{dt} &= f_W W - \frac{\ln 2}{\tau_R} R_W \\ \frac{dR_E}{dt} &= a f_W E - \frac{\ln 2}{\tau_R} R_E \end{aligned} \qquad \text{(S7)}$$

The formal effect is to scale EM viral load by a. This would again lead to cancellation of f_W in the equations for fitting of τ_R from the fraction of WT in resting cells:

$$\frac{d\Lambda}{dt} = W + aE - \frac{\ln 2}{\tau_R}\Lambda$$
$$\frac{db_W}{dt} = \frac{W}{\Lambda} - \frac{W + aE}{\Lambda} b_W \qquad \text{(S8)}$$

In this case we have 2 parameters: τ_R and a, and the estimate of τ_R would depend on the choice of a.

In Table S1 we show the best-fit values for 1 and 2 parameter fits.

Animal	τ_R (1-parameter fit)	$a = f_E/f_W$ (2-parameter fit)	τ_R (2-parameter fit)
19351	<0.5	12.589	<0.5
C0933	<0.5	7.9433	<0.5
25377	<0.5	1.0000	<0.5
2374	<0.5	1.2589	<0.5
C3751	1.3808	1.0000	1.3919
19341	1.7957	1.5849	3.5007
B0526	2.7289	0.12589	<0.5
C5873	2.3105	0.50119	1.8633
B0508	8.1547	0.39811	6.9315
8014	14.623	0.15849	10.502
1.3731	15.861	0.19953	12.378
1335	21.068	0.0100000	5.7762
9183	32.390	0.39811	24.755
8244	30.401	0.19953	19.254
8020	$>10^5$	1.0000	$>10^5$
5424	$>10^5$	0.12589	346.57
9175	$>10^5$	0.0100000	$>10^5$
9021	$>10^5$	0.0079433	$>10^5$

Table S1.

In Fig.S1 we show the estimates of half-life when fitting just τ_R and when fitting τ_R and a simultaneously. There is no significant difference in the medians of the estimated half-lives (although a paired test reveals a significant trend of shorter estimates when we fit both parameters), although the additional parameter improves the fit. In Fig.S2 we show the correlation between half-life and chronic viral load (a) with one-parameter fit and (b) with a two-parameter fit. The same negative correlation holds in both cases with the same degree of significance.

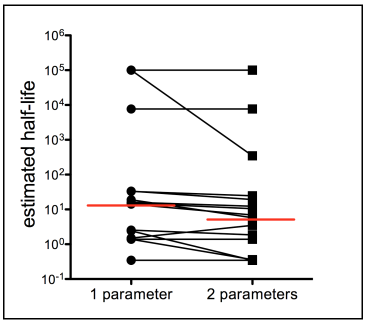

Fig.S1: Half-lives of resting infected cells estimated from one-parameter (Eq.S8, assuming a=1) and two-parameter (Eq.S8) model. Medians are shown in red.

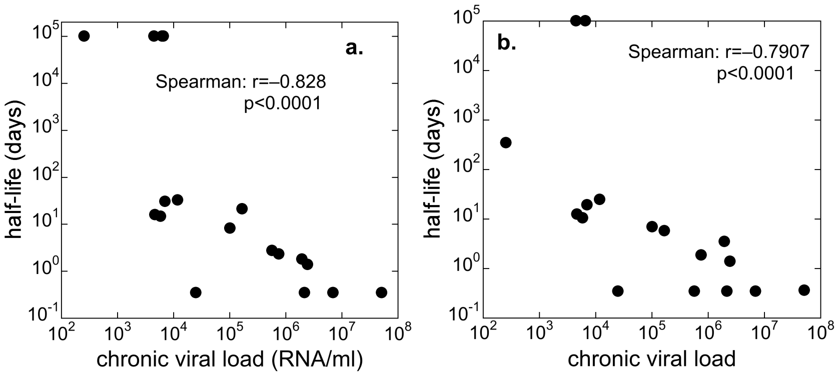

Fig.S2: Correlation between chronic viral load and estimated half-life of resting infected cells; a. fit of just τ_R (Eq.S8, assuming a=1); b. simultaneous fit of τ_R and a (Eq.S8).
